# Stories of change in nutrition in Burkina Faso 1992–2018: a micro-level perspective

**DOI:** 10.1007/s12571-022-01274-z

**Published:** 2022-03-22

**Authors:** Elodie Becquey, Issa Sombié, Mariama Touré, Zuzanna Turowska, Emilie Buttarelli, Nicholas Nisbett

**Affiliations:** 1 International Food Policy Research Institute, Washington, DC USA; 2 Institut Supérieur des Sciences de la Population, Ouagadougou, Burkina Faso; 3 Institute of Development Studies, Brighton, UK

**Keywords:** Burkina Faso, Community perspective, Linear growth, Nutrition, Program

## Abstract

Looking back at what has effectively improved nutrition may inform policy makers on how to accelerate progress to end all forms of malnutrition by 2030. As under-five stunting declined substantially in Burkina Faso, we analyzed its nutrition story at the micro-level. We conducted a regression-decomposition analysis to identify demographic and health drivers associated with change in height-for-age using longitudinal, secondary, nationally-representative data. We triangulated results with findings from semi-structured community interviews (n = 91) in two “model communities” with a history of large stunting reduction. We found that improvement in immunization coverage, assets accumulation and reduction in open defecation were associated with 23%, 10% and 6.1% of the improvement in height-for-age, respectively. Associations were also found with other education, family planning, health and WASH indicators. Model communities acknowledged progress in the coverage and quality of nutrition-specific and nutrition-sensitive sectoral programs co-located at the community level, especially those delivered through the health and food security sectors, though delivery challenges remained in a context of systemic poverty and persistent food insecurity. Burkina Faso’s health sector’s success in improving coverage of nutrition and healthcare programs may have contributed to improvements in child nutrition alongside other programmatic improvements in the food security, WASH and education sectors. Burkina Faso should continue to operationalize sectoral nutrition-sensitive policies into higher-quality programs at scale, building on its success stories such as vaccination. Community leverage gaps and data gaps need to be filled urgently to pressure for and monitor high coverage, quality delivery, and nutrition impact of agriculture, education, and WASH interventions.

**Supplementary Information:**

The online version contains supplementary material available at 10.1007/s12571-022-01274-z.

## Introduction

With only a decade left to end all forms of malnutrition (United Nations, 2015), it is critical that policymakers, government and non-governmental organizations, research and academic bodies and nutrition advocates understand what has already effectively improved nutrition outcomes, and what continues to stymie progress. As evidence is growing on which intervention across several sectors can improve nutrition outcomes (Bhutta et al., 2008, 2013; Keats et al., 2021; Ruel & Alderman, 2013; Ruel et al., 2018), however, there is an acknowledged lack of information on how to effectively operationalize actions, link nutrition interventions to the contexts in which they are situated and to the challenges which they intend to ameliorate, and reach quality and scale, so that action results in tangible nutrition improvements on the ground (Gillespie et al., 2013).

Burkina Faso is an example of a country which has experienced significant improvement in measurable nutrition outcomes for children. After a rise in under-five stunting in the 1990’, Burkina Faso has experienced a regular and consistent decline in stunting since 1998–99 to reach 25% in 2018, resulting in nearly halving stunting rates over the past 20 years (Burkina Faso, 2018; Institut National de la Statistique et de la Démographie, 1994). Improvements in wasting have proved to be more difficult to maintain than in stunting, hovering in the low-teen percentages during the mid-1990s, increasing to 18.6% in 2003 (Institut National de la Statistique et de la Démographie, 2004), but generally declining since then, to percentages between 8.4% and 10.4% since 2014 (Burkina Faso, 2018). Therefore, there is clear indication that nutrition outcomes have improved, but what drove these progresses on the ground is less clear.

This Stories of Change study in Burkina Faso took a community and micro-level perspective with the primary objective of shedding light on the effective drivers and modalities to “on-the-ground" improvements in nutrition in Burkina Faso along with the challenges. By using a narrative approach common to the papers in the Stories of Change series, we are able to integrate the individual experiences described by community members into quantitative analysis on nutrition drivers, thereby forming a larger story on effective drivers of nutrition outcome changes (Bailey, 2015; Gillespie et al., 2016; Gillespie & van den Bold, 2017).

## Methods

This study presents the micro-level perspective of a larger study on stories of change in nutrition in Burkina Faso. A companion study presents findings from the macro-level perspective (Turowska et al., 2022).

This study used quantitative and qualitative methods to examine the trends, dynamics and drivers of change in nutrition in Burkina Faso since 1992, from the micro-level perspective. 1992 was chosen as it was the date of the first International Conference on Nutrition that Burkina Faso attended, which was immediately followed in 1993 by the first Demographic and Health Survey (DHS).

Quantitative methods focused on how nutrition outcomes and expected drivers of nutrition changed over time as well as which drivers were associated with changes in nutrition outcomes. Qualitative interviews were conducted at community level to examine how and why individuals perceived their lives to have changed in terms of livelihoods, food security, nutrition and health.

### Data sources, data collection and management, and data analysis

Table 1 details data sources and methods for data analysis. Briefly, quantitative methods included descriptive analyses and regression-decomposition analyses. Descriptive analyses of secondary data were performed to examine changes in nutrition-relevant drivers since the early 1990s, using data from DHS and National Nutrition Surveys. In addition, to assess which drivers significantly contributed to reduced stunting and improved height-for-age z-scores (HAZ), we used decomposition analysis of data from three rounds of DHS between 1998 and 2010. The data collected as part of the DHS are nationally representative and include a range of standard demographic and health indicators that are consistent across survey rounds, including children’s anthropometric measures and factors known to be associated with children’s health and nutrition status.

**Table 1:** Data sources and data analysis methods

Tool and objective	Data sources	Data/data source selection, data collection and analysis	Profile of data/data source
**Regression analysis**: to identify associations between positive changes in child nutrition over time and concurrent changes in sectoral programs and services	Secondary data analysis from DHS datasets of rounds 1998–99, 2003, 2010 (Institut National de la Statistique et de la Démographie, 2000, 2004, 2012)	• DHS round selection: the 1993 round of DHS was not used as HAZ worsened between 1993 and 1998–99. Since the 2010 round, no further DHS survey has been conducted • Outcome: height-for-age z-scores (HAZ) in children 0–59 mo of age • Selection of potential drivers: Similar studies in other countries (Headey et al., 2017) and the 2013 Lancet framework (Black et al., 2013) informed the selection of proxy indicators that could serve as explanatory variables for changes in HAZ. Those variables available in all three rounds of DHS were included in the analysis as potential drivers • Data analysis: We used multivariate regression analysis pooling the three survey rounds to estimate the marginal effect of the drivers on HAZ, taking village-level clustering into account and controlling for maternal age, child sex and age, and area of residence (rural or urban). We tested regression coefficient differences across rounds and whether coefficients from individual rounds differed from the pooled model, to confirm stability of coefficients. We then used decomposition techniques to calculate the share of change in nutrition outcomes due to each driver by multiplying observed change with marginal effect from regression analyses	Drivers tested (see online resource 1): • Indicators related to feeding and caregiving resources: paternal education (years), maternal education (years), assets index (0–10), number of children • Indicators related to health services: appropriate use of antenatal care (maternal level), child born in a medical facility, age-appropriate immunization • Indicators related to safe hygienic environment: improved drinking water, piped water in residence, improved sanitation facility, open defecation (village level) • maternal height was added to capture the vicious/virtuous inter-generational cycle of malnutrition Potential drivers which could not be tested: • Food security (no consistent longitudinal data) • Infant and young child feeding practices (no longitudinal data) • Malaria control programs (only 2 rounds of data)
**Descriptive analysis**: to describe changes over time in proxy indicators of potential drivers of change in nutrition outcomes	Secondary data analysis from: • DHS datasets of rounds 1993, 1998–99, 2003, 2010 (Institut National de la Statistique et de la Démographie, 1994, 2000, 2004, 2012) • Annual National Nutrition Surveys reports (2009–2018) (Burkina Faso, 2018) • Malaria indicators survey 2014 and 2017–2018 (Institut National de la Statistique et de la Démographie -INSD et al., 2015, 2018)	• Selection of potential drivers and datasets: we focused on the drivers identified for the decomposition analysis (see above) • Selection of datasets: We primarily used all available rounds of DHS. When proxy indicators of pre-selected drivers were not present in the datasets used for the decomposition analysis (see above), we searched for alternative longitudinal datasets or reports available online or through simple request, based on an exhaustive review of national nutrition data sources (Transform Nutrition West Africa, 2019) • Descriptive analysis: we plotted descriptive statistics (prevalence or mean value) of proxy indicators of potential drivers of nutrition available between 1992 and 2018	NA
**Community interviews**: to identify perceived changes in nutrition outcomes and nutrition-related drivers at community levels	Primary data collection through semi-structured in-depth interviews with key informants in the community (n = 79)	• Sampling: Two regions, and within each region, one province, were selected based on their significant reduction in chronic child malnutrition over the past 15 years, and their similarity in terms of climate, with different means of existence. Within each province, 4 villages were randomly selected proportionally to their size (number of households) (n = 8 villages). the 8 village chiefs selected 8 male village leaders, 8 female village leaders, 32 household heads, and 32 wives, who had lived in the village for at least 10 years • Semi-structured in-depth interviews were conducted in 4 local languages (Mooré, Bissa, Nouni, Dagara) or French (as preferred by respondents) by experienced enumerators fluent in the language; digitally recorded; then transcribed in French by the same enumerators with a sub-set of transcriptions reviewed by IS (all languages) and EBe (French). Informed consent was documented at the start of each interview • Verbatim transcripts were coded by research assistants using a pre-defined theme list developed by EBe, EBu and ZT based on the key concepts from our interview guides; a sub-set was reviewed by IS and ZT. Results were summarized in French by research assistants, with spot checks by IS, ZT and EBe, and organized according to the framework presented in Table 2	• One interview of a head of household was not completed because the interviewee had to bring his sick child to the hospital • Respondents profile: Community members were on average 52 ± 12 years old; thirty-three women were housewives and 31 men were family farmers

Qualitative methods comprised semi-structured interviews with key informant community members. Respondents were asked to describe the situation in their village in terms of livelihoods, food security, health and nutrition; how it had changed (positively or negatively) in the past 30 years; and whether they knew some programs, projects, policies, actions, activities or events which positively or negatively impacted food security or nutrition in the past 30 years in their village.

### Triangulation and presentation of findings

Qualitative findings were organized according to a results framework detailed and used in the companion study to triangulate the various stories of change in nutrition in Burkina Faso told from a macro-level perspective (Turowska et al., 2022). The framework outlines 4 components:

1. Leadership and Stakeholders: Who is active/influential/involved in nutrition in Burkina Faso?
2. Ideas, Framing, and Evidence: How is nutrition categorized, perceived, researched, and communicated, in which political and societal context, and how does this affect the nutrition agenda?
3. Institutions, Policy, Coherence, and Accountability: which nutritional programs have been implemented and why, how coordination for executing the programs works, how is accountability ensured, and how do people decide what programs have been successful?
4. Capacity (organizational, financial, systemic, individual), Resources, and Financial Commitments.

The qualitative and quantitative findings were then triangulated and discussed in Sect. 4.

## Results

### Analysis of quantitative data

**Fig. 1:**
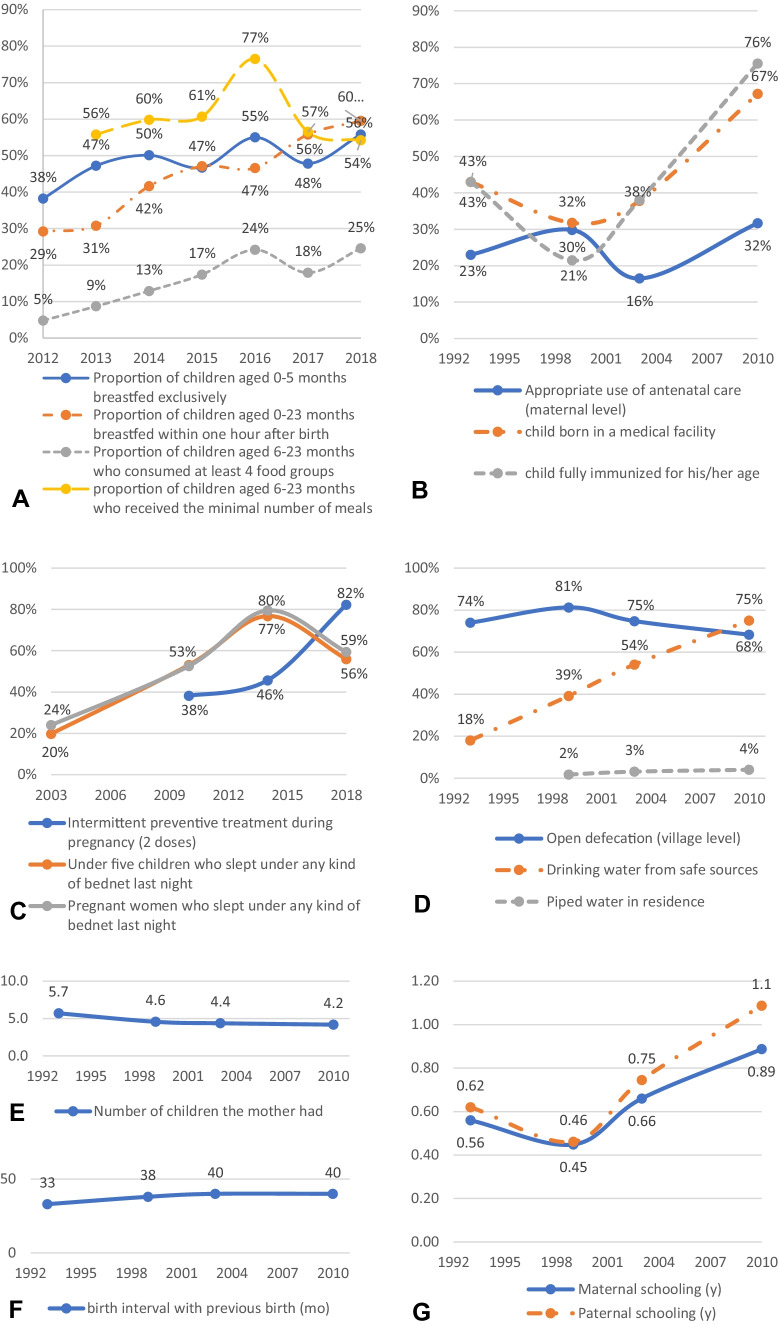
Evolution of proxy indicators for infant and young child feeding programs from 2012 to 2018 (panel** A**), health services from 1993 to 2010 (panel** B**), malaria control programs from 2003 to 2018–19 (panel** C**), water and sanitation programs from 1993 to 2010 (panel** D**), family planning from 1993 to 2010 (panels** E, F**), and education services from 1993 to 2010 (panel** G**). Data sources: Demographic and health surveys 1993, 1998–99, 2003 and 2010 (panels** B, D, E, F, G**), National nutrition surveys 2012 to 2018 (panel** A**), Demographic and health survey 2003 and 2010 and Malaria indicators survey 2014 and 2018–19 (panel** C**)

Between 1998–99 and 2010, the average Height-for-Age z-score (HAZ) improved from -1.74 to -1.38 (Table 2). Results from the regression-decomposition analysis showed that the largest driver of change in HAZ among the drivers considered was improvement in appropriate immunization coverage, which increased from 21% in 1998–99 to 76% in 2010 (Fig. 1B), and was associated with 23% of the improvement in HAZ (Table 2). The second largest driver of change (10% of the improvement) was asset accumulation (measured by an asset index); the third largest driver of change (6.1% of the improvement) was reduction in open defecation at the village level, despite a modest reduction only in open defecation from 81% in 1998–99 to 68% in 2010 (Fig. 1D). We also tested the use of improved sanitation facility at the household level in the model, but it was not significant. The increase from 1998–99 to 2010 in schooling duration of the child’s mother (from 0.45 to 0.89 year) and her partner (from 0.46 to 1.1 year) were independently associated with respectively 1.7% and 2.1% of the improvement in HAZ (Fig. 1G, Table 2). The small but consistent reduction in the number of children the mother had, from an average of 4.6 children in 1998–99 to 4.2 children in 2010, was associated with 1.7% of the improvement in HAZ (Fig. 1E, Table 2). When we ran the analysis on the subsample of lastborn children and included birth interval in the model, birth interval was also associated with a small share of the improvement in HAZ (2.4%, results not shown). The development of piped water in the residence, which increased very slowly from 1.8% in 1998–99 to 4.0% in 2010, was associated with 1.4% of the improvement in HAZ (Fig. 1D, Table 2). However, improvements in drinking water from safe sources was not statistically significant in the multivariable model. Improvement of maternal height by 0.15 cm was associated with 1.8% of the improvement in HAZ (Table 2). Appropriate use of antenatal care for the lastborn child did not show clear improvement with time and was significantly associated with 0.47% of the improvement in HAZ. Finally, 52% of the improvement in HAZ between 1998–99 and 2010 could not plausibly be explained by the drivers tested in the model. Some other drivers could not be tested because of the lack of appropriate data. This included malaria control programs, which showed sharp improvement since 2003, although not sustained from 2014 to 2018 (Fig. 1C); infant and young child feeding programs, which showed moderate improvements since 2012 (Fig. 1A); and food security programs, for which we could not find publicly accessible national longitudinal datasets to use to illustrate or analyze the evolution.

**Table 2:** Proxy indicators for policies and programs associated with improvements in HAZ scores between 1998–2003 and 2010 respective contribution to the progress according to regression-decomposition analysis of DHS data

Variables	Estimate β (1)	Sample mean: 1998–99	Sample mean: 2010	Change in mean (2)	Predicted change in HAZ = (1)*(2)	Share of predicted change in HAZ
HAZ score (outcome)		-1.74	-1.38	0.36	NA	NA
Assets Index (0–10)	0.043	1.6	2.4	0.84	0.036	10%
Maternal education, years	0.014	0.45	0.89	0.43	0.0060	1.7%
Paternal education, years	0.012	0.46	1.1	0.62	0.0074	2.1%
Antenatal care	0.094	0.30	0.32	0.018	0.0017	0.47%
Number of children	-0.016	4.6	4.2	-0.38	0.0061	1.7%
Open defecation (village level)	-0.17	0.81	0.68	-0.13	0.022	6.1%
Piped water in the residence	0.23	0.018	0.040	0.022	0.005	1.4%
Fully immunized	0.15	0.21	0.76	0.54	0.082	23%
Maternal height, cm	0.042	161.5	161.7	0.15	0.0063	1.8%

### Analysis of qualitative data

#### Leadership and stakeholders

In the community, an increasing presence of governmental workers implementing programs was acknowledged by community respondents; this was particularly salient in relation to health programs.

*"Nowadays, community health workers walk around to give malaria drugs to children in households." [Female community member]*

In addition, village authorities gave several examples of how international organizations have affected their lives -referring more to food security than to nutrition. For example, several said that organizations had helped the community build gardens or provided them with materials for gardening and agriculture. There was no direct commentary on which NGOs were responsible for implementing these programs.

Several of the male community members spoke about civil society organizations with which they have had contact, describing producers’ organizations, womens’ groups, and youth groups. These associations were most often described as intended to provide credits, cooperative work, or gain access to farming inputs, not necessarily to improve nutrition. Conversely, the consensus among the female respondents was that associations help improve nutrition, through facilitating women to access credit, which helps them perform income-generating activities, which subsequently allows them to better feed their families:

*"There is the group that helps us to have something to sell so that you can earn and eat" [Female community member]*

#### Ideas, framing and evidence

Among village authorities, a few respondents described what malnutrition looks like, citing slimming of the body, kwashiorkor, faded skin, and weakness. The majority defined malnutrition as a disease linked to insufficient food in quantity and quality; in addition, several of them linked malnutrition to detrimental hygiene, health, or care practices.

*“For example, a woman who is breastfeeding and before her child is healthy, or starts to walk, she becomes pregnant prematurely, this can cause illness in the child.” [Village authority]*

They generally said that malnutrition stems from several issues including unemployment, lack of financial means/poverty, lack of education in nutrition issues, poor quality food, and sickness. Many of the authorities mention the role of women in preventing malnutrition, as they are the ones who typically give food to children. A few respondents commented that traditional beliefs about child-feeding still affect women’s decisions in feeding children, though less than it used to.

*“At the beginning there were a number of taboos: the child should not eat the eggs; the child was not to eat beans etc., which meant that the child was deprived of certain nutrients in his diet. These taboos were under the pretext that if the child ate these foods, he would become a thief in the future…For example a pregnant woman needs a balanced diet, but with ignorance she is reproached that these foods are forbidden for a pregnant woman.” [Village Authority]*

Among the household heads and their wives, the consensus was that malnutrition is a disease caused by lack of food or vitamins. Many of them emphasized that children in particular are vulnerable to becoming malnourished. The vast majority were aware of malnutrition and were able to describe causes or consequences. It appeared that community members were able to access nutrition information through radio broadcasts.

#### Institutions, policy, coherence, and accountability

Respondents acknowledged being increasingly reached over the past decades by programs from multiple sectors which have impacted their nutrition by providing greater access to nutrition services for prevention and treatment of malnutrition, greater counselling on child feeding and nutrition, greater access to fortified and nutritious foods, greater access to the market, and greater safety nets such as access to shops selling subsidized staple foods through the société nationale de gestion des stocks de sécurité alimentaire (SONAGESS).

*"Nowadays, the mother of the child is offered chocolates [local name for lipid-based nutrient supplements], flours for porridge, and in addition to that, we give the mother advice concerning the child" [Village Authority]*

Some of the respondents reported that gardening is a means for supplementing diet in the lean season and described projects/programs for developing market gardens. Increased involvement of women in income-generating activities were also reasons cited for improved nutrition.

*"They [women] go to market gardens, they buy eggplants and all kinds of food that they come to sell in the market. At the end of the day, they use the profit to prepare the sauce they want to eat. So it's not like before.” [Male community member]*

Other drivers of change in nutrition spontaneously cited by the community include an increase in the number of girls attending school, an increase in time intervals for women between pregnancies, increased planning of birthing dates, decreased rates of neonatal mortality, safer deliveries for women. Many respondents mentioned that women and children below 5 years old receive free healthcare. Free healthcare has also positively affected food security because in the past community members were forced to sell farmed foodstuffs to care for children. Some respondents said there has been increased access to vaccination and malaria control programs, and that health workers are increasingly present. It came clearly that women and children generally are the target groups for health interventions.

*“Now, care for children and pregnant women is free. Similarly, mosquito nets are given to pregnant women and one show them how to feed the baby. In the old days, women gave birth at home, where there were risks. Now they go to give birth at the maternity ward. The health center came to save us.” [Male community member]*

*"Before there were diseases like polio that prevented children from growing well and one met many children with disabilities. But nowadays, there are many diseases that are no longer visible in our village." [Male community member]*

A few respondents also said that there has been a shift away from traditional healing techniques. However, a small minority were skeptical of modern medicine:

*"Today's children you see they are beautiful, but they are not healthy, but before here, as I said we boiled roots, we washed and purge but today it's shots” [Male community member]*

Despite these improvements, several respondents also mentioned barriers to accessing potentially helpful services. For example, a few respondents complained about health centers being too far to reach, costs of drugs to be too high, drugs being unavailable, or high costs of transportation to trainings or health centers. Also, some respondents stated that although there has been some increase in gardening, at the same time there has also been movement away from sorghum and millet and into maize and rice production, and associated changes in traditional diets, including shifts in consumption toward less micronutrient-dense staple crops, and increased availability and overconsumption of non-nutritious foods.

*"Before we ate tô [a flour-based staple dish] made from millet or red sorghum, but today, the children do not eat it, they want the tô from corn.” [Male community member]*

*"At present it is more difficult than before because before there were only pancakes and now there are cakes, donuts and there is a lot of food today" [Female community member]*

#### Capacities, Resources, and Financial Commitments

The majority of community members described a positive systematic shift in access to healthcare in recent years and described improvements in healthcare systems due to increases in health human resources, free care for women and children under five, and modernization of services; yet community members said that they continue to face challenges in reaching health centers, in having means of communication to alert health authorities in case of emergency, and in paying for expensive prescriptions.

*“If we thought about children we would build a health center. From here to [village with health center], is how many kilometers?” [Female Community member]*

In addition, the general consensus among respondents was that there has been a positive evolution in access to food, as well as a greater variety in the foods available, because the food system has improved, i.e. producers have diversified the crops they farm and are able to sell their harvests for money to buy other produce.

*"What women also earn, if it comes as a supplement, it will not be only the staple food that we eat every day, what they have can come in and alternate the menu" [Male community member]*

*“We buy from the stores. There are foods that comes from other places like Ghana.” [Male community member]*

Agriculture remains the main activity that allows people to feed themselves, yet many respondents mentioned that it is necessary to have a supplemental income-generating activity (trade, fishing, mining, carpentry, masonry, welding, mechanics, scrap metal, brick making, selling wood, sewing, and driving were cited) in order to have money to buy food, because agriculture is “like a lottery.” Many respondents said that harvests no longer last through the lean season, and people are forced to purchase food at the market or from the “SONAGESS” (selling subsidized foods). Although food is available in markets, the constraints producers face in production eventually determine if they can afford to buy other food. Several of the respondents also made complaints about the new paradigms surrounding food access, including increased food costs and manipulative purchasing behaviors:

*“During this harvest period, those who have money buy the grain from us at a good price. Then, when the lean season come, they sell us our own cereals at elevated prices” [Male community member]*

Respondents spoke about how communities rely on government structures to deliver and provide access to agricultural inputs like fertilizer, improved seeds, and machinery. However, farming support systems like extension services, infrastructures, and subsidies for inputs do not reach producers at scale.

*“The land is weak here, there is not even someone to come show us how to cultivate, how we should do it or how you can arrange your land so that the water does not wash away the little fertilizer that is in your soil.” [Village Authority]*

*"There is no dam to do market gardening.” [Male community member]*

In general, respondents reported population growth and the pressures it puts on employment opportunities.

Regarding women’s empowerment, community members said that women were systematically disadvantaged because they cannot own land, yet actions like increased credit access, training/educational talks for women, increased opportunities for income-generating activities, and free healthcare were increasingly visible. This allowed women to better care for themselves and their households.

*“The village can only advance forward ... Women are always taken into account in projects, programs and involved in agricultural activities” [Male community member]*

*“Nowadays, the wife and her husband are both involved in feeding the family. Before, it was the man who went out looking for the family but that is no longer the case. They [women] go out in the morning like their husbands and only come home in the evening with the sole purpose of having something for the family.” [Village Authority]*

Regarding individuals’ capacities, community members said that at the individual level, poverty persists, and lack of money hinders access to nutrition drivers such as education (they stated that schooling fees and materials are expensive), diversified food, and agricultural inputs. Respondents had differentiating experience in whether their incomes had fallen or increased. Overall, respondents said that communities lack sufficient leverage to demand financing from the government. Finally, respondents described chronic problems like cyclical food crises and pests which continue to plague communities.

## Discussion

Burkina Faso quantitatively documented noticeable progress in the coverage of several programs across the health, education and WASH sectors since 1992, which were significantly associated to improvements in child linear growth from 1998–99 to 2010. These included very specific sectoral programs such as vaccination, reduction in open defecation, education, piped water, family planning and antenatal care. Community interviews conducted in 2018 in two model provinces confirmed these and other programs across the health, agriculture and education sectors have increasingly reached communities. These other programs included free healthcare to pregnant women and children under five, malaria control, community management of acute malnutrition, breastfeeding counselling, and programs to improve access to fortified complementary foods, subsidized foods, and micronutrient-dense foods through gardening and markets. Overall, community respondents credited the tangible progresses they did experience relative to child and maternal health and nutrition to these programmatic improvements.

Health was the sector whose success was the most salient in our findings, across and within our qualitative framework items, and the quantitative analysis. Successful health programs which emerged included pure nutrition programs, but also preventive health, treatment of disease and family planning. Immunization is an example of a nutrition-sensitive health policy effectively translated into a program implemented at scale. Immunization was found to be critical for reducing stunting in our quantitative analysis and was spontaneously cited by community members as having led to visible change. Immunization prevents common childhood illnesses and, as such, impacts child health status and nutrition. Child immunization is fully integrated into the national health policy (Burkina Faso, 2017a) and implemented at scale free of charge to families through a national program. The latest statistics on appropriate immunization coverage suggest that universal coverage has nearly been reached in Burkina Faso for most childhood vaccines (WHO & UNICEF, 2017), which might be associated to further improvements in nutrition since 2010. Antenatal/perinatal care is another example of health service found to be associated to improvements of child linear growth and for which improved access and utilization was acknowledged by communities, although with remaining challenges. Burkina Faso is currently revising its national policies and programs related to antenatal care, to include some of the most recent WHO recommendations for antenatal care for a positive pregnancy experience (World Health Organization, 2016). Future significant improvements in appropriate antenatal care coverage, if they exist, might result in measurable impact on intra-uterine and child growth in Burkina Faso. Community members also mentioned the reach of malaria control programs, but we lacked data to test the association between the introduction and extension of malaria control programs and child linear growth, although we showed these programs gradually reached scale since introduced on the early 2000’. Anecdotal evidence in Burkina Faso showed that malaria in children was a large, direct determinant of child anemia (Bliznashka et al., 2020). Communities also acknowledged a positive experience with nutrition programs for which we had no quantitative data available, including the scale-up of community management of acute malnutrition, breastfeeding counselling, and access to fortified complementary foods. This appears to concur with a rigorous mapping exercise at the country level which revealed that many nutrition-specific interventions had satisfactory geographical coverage in 2014 (Doudou et al., 2018). Free healthcare to pregnant women and children under five was largely cited by community members as a very recent driver of tangible improvement, and has proven to lead to a significant 17% increase in the use of health services in the longer term (Zombré et al., 2019). Finally, family planning programs that aim at optimizing age at first pregnancy, family size and inter-pregnancy intervals, lead to better health and nutrition pregnancy outcomes (Bhutta et al., 2013); There is currently a large, though declining, unmet need for contraceptives in Burkina Faso, especially for spacing births (Institut Supérieur des Sciences de la Population, 2018). Our findings suggest their expansion might lead to further measurable positive impacts on child linear growth.

Besides the health sector, programs from three sectors–Agriculture, Education and WASH–emerged from our quantitative and/or our qualitative findings. Unfortunately, we lacked data to test association between changes in food security or agriculture programs and nutrition outcomes: indicators of food security have not been included to date in the Burkina Faso DHS, and the annual national permanent agriculture survey and nutrition survey are distinct in Burkina Faso. Nevertheless, model communities acknowledged a positive experience with access to subsidized foods, and access to an increasing variety of micronutrient-dense foods through gardening and improved access to markets, although they also cited numerous challenges and gaps in the implementation of agriculture programs.

Our quantitative analysis showed that small improvements in paternal education and maternal education were independently associated to improvements in child nutrition, and communities cited improved health and nutrition knowledge, as well as improvements in girls’ schooling, as drivers of nutrition changes. Evidence from a multi-country analysis showed that parental education does have nutrition returns in the next generation, but are modest, and limited by low quality of education services (Alderman & Headey, 2017). Also, returns are much higher when a critical schooling level (secondary) is attained. So, the small improvements seen here, even given Burkina Faso’s low base level of educational years completed, show that there are potentially untapped gains for stunting still to be achieved via improving access to education.

Proxy indicators for national programs and services in the WASH sector (access to piped water, and prevalence of open defecation) also showed measurable improvements and were significantly associated to some extent to improved nutritional outcomes, in spite of little progress in national coverage, and virtually no evidence in the two model provinces of community acknowledgement of WASH programming or awareness of the potential association between WASH programs and nutrition progress. Poor drinking water quality directly impacts the nutritional status through the ingestion of contaminated material including pathogenic bacteria and nematode eggs, leading to diarrhea, environmental enteropathy and nematode infection (Dangour et al., 2013). Interventions to improve water quality for preventing diarrhea are the most efficient when they provide a safe storage container (Clasen et al., 2015), probably because inappropriate water storage in poor hygienic environments can lead to re-infestation of safe water. Inadequate water storage seems to be the norm in Burkina Faso, with most drinking water stored in jars without a tap, often uncovered or with no fitted cover, sometimes visibly dirty, and in which any kind of vessel is regularly immersed to retrieve water (Becquey et al., 2014; Ngure et al., 2019). This might explain why improvements in drinking water from the pipe only (not from other safe sources) was significantly associated to child linear growth. Also, our findings are consistent with the knowledge that sanitation programs should aim at mobilizing entire communities – not just individual households–to become free of open defecation (Cronin et al., 2017). Programs such as community-led total sanitation have been shown to reduce stunting by 6 percentage points in rural Mali (Pickering et al., 2015). Given their association with child linear growth, the slow historical improvements in their coverage and the lack of community awareness of their potential impact on health and nutrition, the expansion of WASH programs should be a high priority in Burkina Faso and may require strong behavior change mechanisms.

Across sectors, it came largely from our findings that inclusion has been and will likely continue to be key to success, namely the inclusion on women in agriculture and income generating activities, granting education to women, enabling women to access health services, access credit and own land, and creating opportunities for youth. This finding is supported by experimental evidence from Burkina Faso which showed that women’s empowerment led to higher returns on child wasting of a nutrition-sensitive agriculture program (Heckert et al., 2019).

Our findings described a co-location at the community level of nutrition-specific and nutrition-sensitive sectoral programs and actions across sectors, consistently related–quantitatively and qualitatively- to positive nutrition outcomes. It is still uncertain whether multisectoral programming, besides co-location of services, is required to ensure larger impact. Simple tools such as multisectoral mapping proved to be efficient in identifying gaps in nutrition-sensitive interventions coverage and funding and in engaging stakeholders, and have been recommended in Burkina Faso (Doudou et al., 2018). Although we showed that several programs (e.g. immunization) might have impacted nutrition without specifically aiming at doing so, integration of nutrition into sectoral policies may bring synergistic returns on both nutrition and sectoral outcomes. For example, nutrition services at school may provide an incentive to improve schooling coverage and duration and may improve school performance. Burkina Faso’s multisectoral nutrition plan 2017–2020 (Burkina Faso, 2017b) and 2020–2024 (Burkina Faso Ministère de la Santé, 2020) include proposals for nutrition-sensitive sectoral and multisectoral actions, and these should be implemented.

A main limitation of our study included the lack of integrated quantitative data: particularly, we were missing integrated data on recent programs such as community management of acute malnutrition, breastfeeding counselling, access to fortified complementary foods, malaria control, food security programs or nutrition-sensitive agriculture programs. Also, data were old, and though a recent round of DHS survey was eventually organized in the end of 2019, neither the report nor the micro-data were publicly available 2 years later. Finally, the designs of neither our quantitative nor our qualitative study allow to firmly credit the change in nutrition observed in Burkina Faso to the changes in nutrition programming observed in survey data or acknowledged in the field. However, the triangulation of consensual findings from mixed methods reinforces the strength of this possible explanation. Also, evidence is strong that several of the programs identified through our study are effective to improve nutrition, including recent evidence on malaria prevention, preconception care, and water, sanitation, and hygiene promotion delivered inside and outside the health-care sector (Keats et al., 2021). Therefore, it is plausible that the success of Burkina Faso in developing the coverage, and possibly the quality of implementation of these programs, resulted in the consistent positive changes observed in nutrition outcomes.

Overall, the ground-level stories of change reported in this manuscript draft a picture remarkably consistent with the macro-level perspective presented in our companion study, which identified success stories in creating an enabling environment for nutrition in Burkina Faso between 1992 and 2018, from a policy and central level perspective (Turowska et al., 2022): the leadership of the health sector; the effective cooperation between actors in nutrition, with a strong leadership and financial contribution of international actors, and rising influence of the civil society; the increasing sectoral and multisectoral nutrition awareness and cooperation, historically with the food security sector, and increasingly with other sectors; and sectoral progresses in translating the central level policy structure into sectoral action at scale, especially in the health and agriculture sectors. Together, the stories from the macro and the micro-level suggest that Burkina Faso nutrition strategy has been effective and could be a model for other countries. Moving forward, Burkina Faso should continue to operationalize sectoral nutrition-sensitive policies into effective programs at scale, building on its successes stories such as vaccination, while ensuring through integrated data and monitoring the high coverage, quality delivery, and nutrition impact of agriculture, education, and WASH interventions.

## Supplementary information

Below is the link to the electronic supplementary material.

Supplementary file1 (DOCX 14 KB)

## Data Availability

We used publicly available quantitative datasets. Primary qualitative data (interviews) cannot be de-identified.

## Code availability

Stata syntaxes are available upon request.
