# Supplementary material for: Stories of change in nutrition in Burkina Faso 1992–2018: a micro-level perspective
Source: Food Secur. 2022 Mar 22;14(4):937–50. doi: 10.1007/s12571-022-01274-z (PMC9325828; doi:10.1007/s12571-022-01274-z)
Supplement: Supplementary file 1 — Supplementary file1 (DOCX 14 KB) [file 12571_2022_1274_MOESM1_ESM.docx]

**Stories of change in nutrition in Burkina Faso 1992-2018**

**Online ressource 1. Overview of proxy indicators available in DHS for potential drivers of change in child linear growth**

**Feeding and caregiving resources**

*Paternal education and maternal education (years) -* The number of years of schooling achieved was used for both the mother and her partner.

*Assets index* - We built an assets index (between 0 and 10) by calculating loadings on the first component of a principal component analysis, considering possession of the following assets: bicycle, radio, car or motorbike, improved floor (other than earth/sand/planks), TV set or fridge, electricity.

*Number of children* – We used the total number of children ever born from the mother as an indicator of fertility.

*Birth interval -* The interval between a child birth and the birth of the previous child was collected only for children who were the most recently born from their mother, leading to the non-random exclusion of 18% of the children (mostly in the oldest age ranges). In addition, birth average interval increased by only 2 months over a decade (from 38 months in 1998-99 to 40 months in 2010). Therefore, we excluded this variable from our analysis.

**Health services**

*Appropriate use of antenatal care (maternal level) –* This indicator was defined as receiving the recommended 4 antenatal care visits at the health center during pregnancy. Individual data on appropriate use of antenatal care was collected only for the most recent child. Considering this practice only during the pregnancy of each individual child would have led to the non-random exclusion of 21% of the children (mostly in the oldest age ranges). We therefore used the indicator value for the last child as a proxy indicator for maternal use of antenatal care for all her under-five children.

*Child born in a medical facility* – Whether the child was born in a medical facility was used as a proxy indicator for the individual access to and utilization of perinatal health services.

*Age-appropriate immunization* – We calculated age-appropriate immunization using the following immunization schedule: 1 month of age, Polio0 + BCG; 3 months, previous vaccines + DPT1 + Polio1; 4 months, previous vaccines + DPT2 + Polio2; 5 months, previous vaccines + DP3 + Polio3; 12 months, previous vaccines + Measles.

**Safe hygienic environment**

*Improved drinking water* - Drinking water was considered improved if coming from the following main sources, regardless of water storage: tap, borehole, protected well or rain.

*Piped water in residence* – Safety of drinking water storage was not collected in any survey round. We identified piped water in residence as the only source of drinking water which would not require storage, hence not compromise the potability of water from a safe source by inadequate storage.

*Improved sanitation facility -* Sanitation facility was considered improved if connected to a drain or pit and equipped with a flush, stab or ventilation.

*Open defecation (village level) -* Open defecation impacts hygiene of the environment and has detrimental consequences at the community level. We calculated the proportion at the village level of children who lived in a household with no sanitation (whether improved or not).
